# A novel nomogram to predict the reliability of estimated glomerular filtration rate formulae in oncology patients

**DOI:** 10.1186/s12885-020-06997-w

**Published:** 2020-06-08

**Authors:** Yichun Cheng, Liu Huang, Yunfeng Han, Chummun Vanisha, Shuwang Ge, Gang Xu

**Affiliations:** 1 Department of Nephrology, Tongji Hospital, Tongji Medical College, Huazhong University of Science and Technology, 1095 Jiefang Ave., Wuhan, P. R. China; 2 Department of Oncology, Tongji Hospital, Tongji Medical College, Huazhong University of Science and Technology, Wuhan, P. R. China; 3 Department of Nuclear Medicine, Tongji Hospital, Tongji Medical College, Huazhong University of Science and Technology, Wuhan, P. R. China

**Keywords:** Estimated glomerular filtration rate, Measured glomerular filtration rate, MDRD formula, Nomogram

## Abstract

**Background:**

Formulae of estimated glomerular filtration rate (eGFR) based on serum creatinine (Scr) are routinely used in oncology patients, however, they are inaccurate in some populations. Our aim was to assess the agreement of eGFR formulae and thereby build a nomogram to predict the reliability of estimates.

**Methods:**

Measured GFR (mGFR) using isotope from 445 oncology patients were compared with eGFR from six formulae (Cockcroft-Gault, Modification of Diet in Renal Disease (MDRD), modified MDRD formulae for Chinese (C-MDRD), Chronic Kidney Disease Epidemiology (CKD-EPI) Collaboration, Wright and full age spectrum (FAS)). Bias, precision and accuracy of eGFR formulae were examined. We also evaluated statistics of agreement: the total deviation index (TDI), the concordance correlation coefficient (CCC) and the coverage probability (CP). Multivariate logistic regression was applied to identify characteristics associated with inaccurate eGFR and construct a predictive nomogram.

**Results:**

All eGFR formulae tended to overestimate the eGFR. The percentage of patients with eGFR within 30% the mGFR ranged from 38.0 to 62.8%. Cockcroft-Gault and MDRD showed low bias and high precision. The MDRD formula exhibited lowest TDI, meaning that 90% of estimations ranged from − 36 to 36% of mGFR. Multivariate logistic regression showed that inaccuracy of MDRD was found in elderly patients or in patients with eGFR greater than 120 ml/min. A nomogram was constructed to help oncologists to predict the risk of inaccuracy of eGFR. The calibration curve showed good agreement.

**Conclusions:**

Our results suggest that the error of eGFR by any formulae was common and wide in Chinese oncology patients. Our nomogram may assist oncologists in decision-making when mGFR is needed.

## Background

Accurate assessment of the glomerular filtration rate (GFR) is important in drug dosing, decision making and assessing the prognosis of oncology patients. Radioisotopic methods have been used as accurate GFR measurements in clinical practice [1, 2]. However, these methods are relatively costly, time consuming and require blood sampling. As a substitute, several formulae have been developed to calculate the estimated glomerular filtration rate (eGFR) based on serum creatinine (Scr) concentration, as well as on age, sex and weight of the patient [3–8].

There have been some reports about the accuracy of these creatinine-based eGFR formulae in oncology patients. However, most of them showed that the performance of the eGFR formulae were unacceptable and thus the precision of chemotherapy dosing was low in some oncology patients [9–11]. Such inaccuracy may result in severe side effects, including death. Therefore, it is necessary to recognize the population where eGFR formulae are unreliable and alternative GFR measurements should be employed. Some studies indicated that creatinine-based eGFR formulae were less accurate in elderly and obese populations [12–14]. However, a limited number of studies were based on an oncology population [14]. Furthermore, to our best knowledge, none of these studies took into account other potential confounding factors, such as sex, nutritional status and comorbidities.

To bridge the gap in current knowledge, we determined to evaluate the performance of the Cockcroft-Gault [3], Modification of Diet in Renal Disease (MDRD) [4], modified MDRD formulae for Chinese (C-MDRD) [6], Chronic Kidney Disease Epidemiology (CKD-EPI) Collaboration [7], Wright [5] and Full Age Spectrum (FAS) [8] formulae in estimating GFR compared with the measurement of the GFR using technetium-99 m diethyl triamine penta-acetic acid (^99m^Tc-DTPA). More importantly, we aimed to identify the characteristics influencing the accuracy of eGFR formulae. A clinically applicable nomogram was constructed to recognize the populations at high risk of inaccurate eGFR and thereby measurements of GFR should be used.

## Methods

### Patients

We enrolled oncology patients with histologically confirmed and measured GFR (mGFR) by ^99m^Tc-DTPA in the Tongji Hospital of Huazhong University of Science and Technology (Wuhan, China) from January 2013 to December 2016.

Patients younger than 18 years old or with missing information were excluded. Patients with acute kidney injury or on any kind of renal replacement therapy were also excluded. Finally, the population consisted of 445 patients.

### Laboratory method and GFR calculations

GFR was measured by radioisotopic method using ^99m^Tc-DTPA, which was reported to had virtually identical clearances with inulin and can be used for measuring GFR [15, 16]. All procedures were performed at the Nuclear Medicine Department of Tongji Hospital of Huazhong University of Science and Technology. Patients were hydrated with 300 ml of water 30mins before the examination. Radioactivity of the syringe containing ^99m^Tc-DTPA was counted before injection. Then, patients were given a bolus of intravenous injection of approximately 185 MBq DTPA into the forearm and the dynamic renal flow images were collected immediately. The post-injection syringe was also counted. The exact dosage of administered ^99m^Tc-DTPA was defined as the difference of the syringe’s radioactivity between pre- and post-injection. The calculation of GFR values was done by the Xeleris™3 Functional Imaging Workstation automatically according to the modified Gate’s equation [17]. The mGFR was reported as ml/min.

Serum creatinine was measured by Roche enzymatic assay (Shanghai Roche Diagnostic Products Co, Ltd., China) within a week before measurement of GFR. The last one was chosen for patients with multiple records. We selected eGFR formulae that had been applicated widely or derived from cancer patients. Although Cockcroft–Gault [3] and MDRD [4] formulae were developed in western population, it has been widely applicated in clinical routine in China for past decades. In recent years, CKD-EPI formula [7] was derived in a large dataset and recognized widely in China. Besides, Wright formula [5] was developed from cancer patients. We also validated C-MDRD formulae, which was designed for Chinese population [6]. FAS formula was developed for full age spectrum in 2016. However, we cannot obtain appropriate Q-value for Chinese population from previous studies, thus, we added validation of FAS formula by introducing Q-value listed in the original publication [8]. The details of formulae used in this study are presented in Table 1.

**Table 1:** Calculations used

	Equations
BSA (m^2^) [DuBois]	0.007184 ×Weight^0.425^ × Height^0.725^
BMI (kg/m^2^)	Weight (kg)/Height(m)^2^
Cockcroft-Gault (ml/min)	$\frac{\left(140-\mathrm{Age}\right)\times \mathrm{Weight}}{72\times \mathrm{Scr}}$ (× 0.85, if female)
MDRD (ml/min)	175 ×SCr^-1.154^ ×Age^-0.203^ × $\frac{\mathrm{BSA}}{1.73}$ × (0.742, if female)
C-MDRD (ml/min)	175 ×SCr^-1.234^ ×Age^-0.179^ × $\frac{\mathrm{BSA}}{1.73}$ × (0.79, if female)
CKD-EPI (ml/min)	141 ×min($\frac{\mathrm{Scr}}{\upkappa}$ ,1)^α^ × max($\frac{\mathrm{Scr}}{\upkappa}$ ,1)^-1.209^ × 0.993^Age^ × $\frac{\mathrm{BSA}}{1.73}$ (× 1.018,if female)(× 1.159,if black) Note: κ is 0.7 for females and 0.9 for males, α is− 0.329 for females and − 0.411 for males.
Wright (ml/min)	$\frac{\left(6230-32.8\times \mathrm{Age}\right)\times \mathrm{BSA}\times \left(0.77\;\mathrm{if}\kern0.17em \mathrm{female}\right)}{\mathrm{Scr}\times 88.4}$
FAS (ml/min)	For 2 ≤ age ≤ 40 years: 107.3/ (Scr/Q) × $\frac{\mathrm{BSA}}{1.73}$ For age > 40 years: 107.3/ (Scr/Q) × (0.998)^(Age-40)^ × $\frac{\mathrm{BSA}}{1.73}$

*BSA* body surface area (m^2^), *BMI* body mass index, *GFR* glomerular filtration rate, *Age* age in years, *Weight* weight in kilograms, *SCr* serum creatinine (mg/dl), *MDRD* modification of diet in renal disease, *C-MDRD* modified MDRD Study equation for Chinese, *CKD-EPI* chronic kidney disease epidemiology collaboration, *FAS* full age spectrum. In order to express all GFRs in mL/min, we de-indexed eGFR for body surface area

### Statistical analyses

Continuous variables are described as mean and standard deviation (SD), with univariate comparisons performed using the Student’s t-tests. Categorical variables were assessed by χ2 or Fisher’s exact test, as appropriate.

The degree of bias for each formula was quantified by percentage error (PE) between the eGFR and mGFR, that is, (eGFR-mGFR)/mGFR in %. Precision was assessed by absolute percentage error (APE), that is, the absolute value of PE. Accuracy was assessed as the percentage of patients with within 30 and 10% of the mGFR (P30 and P10). The agreement between eGFR and mGFR was also assessed by specific statistics for continuous data, including the concordance correlation coefficient (CCC), total deviation index (TDI), coverage probability (CP) [18, 19]. We defined a priori that acceptable bias between eGFR and mGFR should be at least 10%, and that 90% of the estimations should be included within these limits. Bland-Altman plots were used to study the relationship between the difference between eGFR and mGFR and the mean of both [20].

We used logistic regression to find the risk factors of inaccurate eGFR, which was defined as the eGFR outside 30% of mGFR. To be able to construct a prediction model that is clinically relevant while also being simple to use, categorization were performed for continuous variables. We used the cut-points which were widely recognized and adopted in previous studies or the quantile. The significant factors on univariate logistic regression analysis along with clinically relevant factors were entered into a multivariate logistic regression analysis. Backward selection based on the Akaike information criterion (AIC) was used to filter out factors that were entered into a predictive model [21]. The final model equation was then organized as a nomogram. Discrimination of the nomogram was assessed using the area under the receiver operating characteristic curve (ROC). Calibration was assessed using a calibration curve. Given that the predictive model tends to be overfitted to the original sample, a bootstrapping resampling (200 repetitions) was used for internal validation to obtain relatively unbiased estimates.

Statistical analyses and were performed with R version 3.4.0. For agreement analyses, we used a software (AGP Agreement Program v1.0 IGEKO, SP) available at: www.ecihucan.es/lfr/apps/?dir=agreement_installer [22]. All tests were two-sided and *P* < 0.050 was considered statistically significant.

## Results

### Patient characteristics

General demographic and clinical characteristics for the 445 identified patients are given in Table 2. Mean age was 57 ± 12 years old and 66.7% patients were women. The mean mGFR was 68 ± 21 ml/min.

**Table 2:** Patients Characteristics

Characteristic	Mean ± SD	Range
Sex, male, n (%)	296 (66.7)	
Age	57 ± 12	23–88
Weight (kg)	64 ± 11	35–93
Height (cm)	165 ± 8	142–185
BSA (m^2^)	1.71 ± 0.16	1.27–2.17
BMI (kg/m^2^)	23.4 ± 3.2	14.2–32.0
Albumin (g/L)	38.5 ± 4.6	23.9–69.3
BUN (mmol/L)	5.9 ± 2.0	1.6–14.0
Scr (μmol/L)	88 ± 37	39–386
BUN/Scr ratio	17.6 ± 6.0	4.5–53.0
Hemoglobin (g/L)	126 ± 22	43–188
mGFR (ml/min)	68 ± 21	12–137
Diabetes, n (%)	44 (9.9)	
Hypertension, n (%)	140 (31.5)	
Metastasis, n (%)	49 (11.0)	
Obstructive nephropathy, n (%)	104 (23.4)	

Data are presented as the mean ± SD and *N* (%). *BSA* body surface area, *BMI* body mass index, *BUN* blood urea nitrogen, *Scr* serum creatinine, *mGFR* measured glomerular filtration rate

### Performance of eGFR formulae

**Fig. 1:**
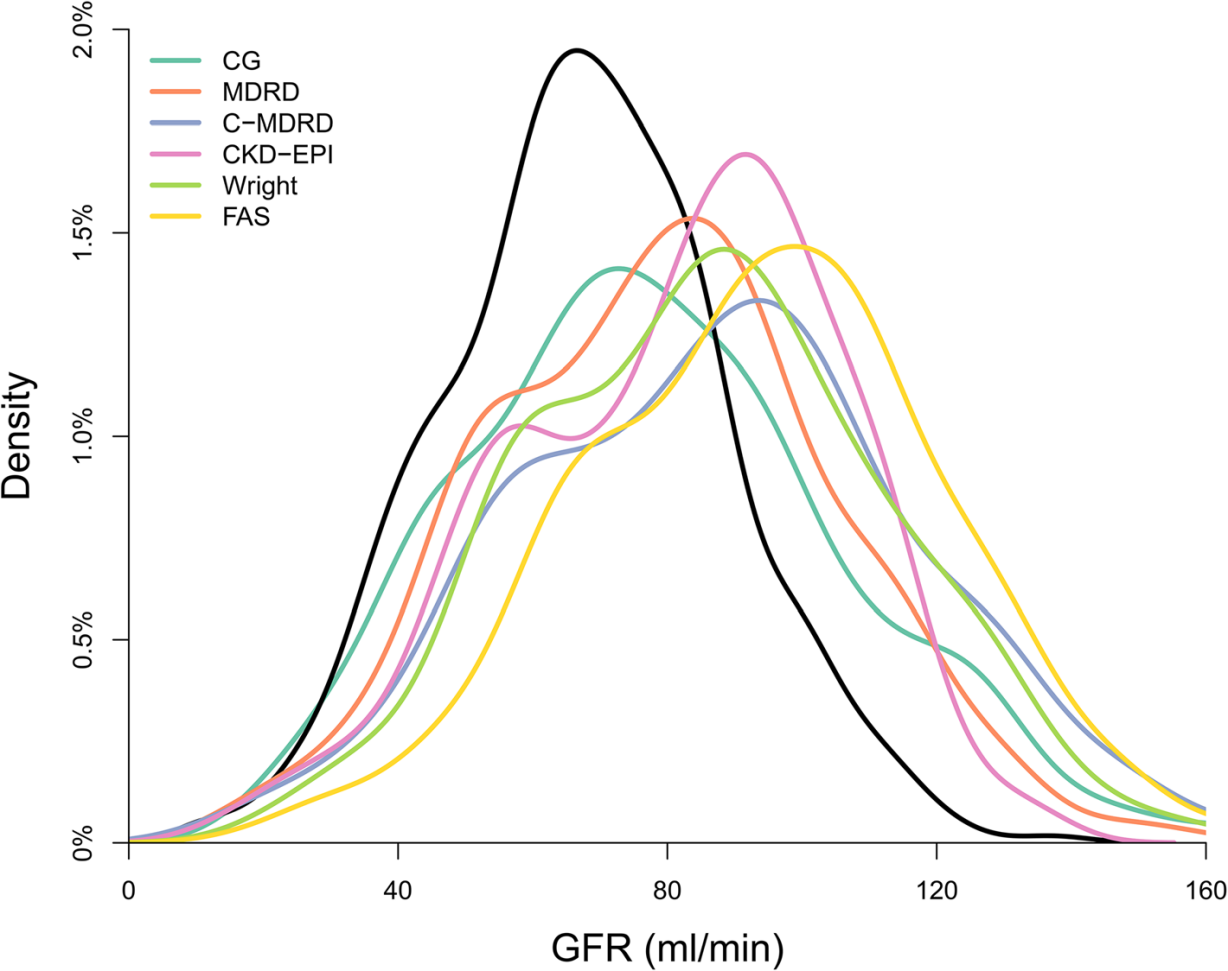
Distribution and CKD prevalence of estimated and measured GFR. Distributions are demonstrated using kernel density plots

**Fig. 2:**
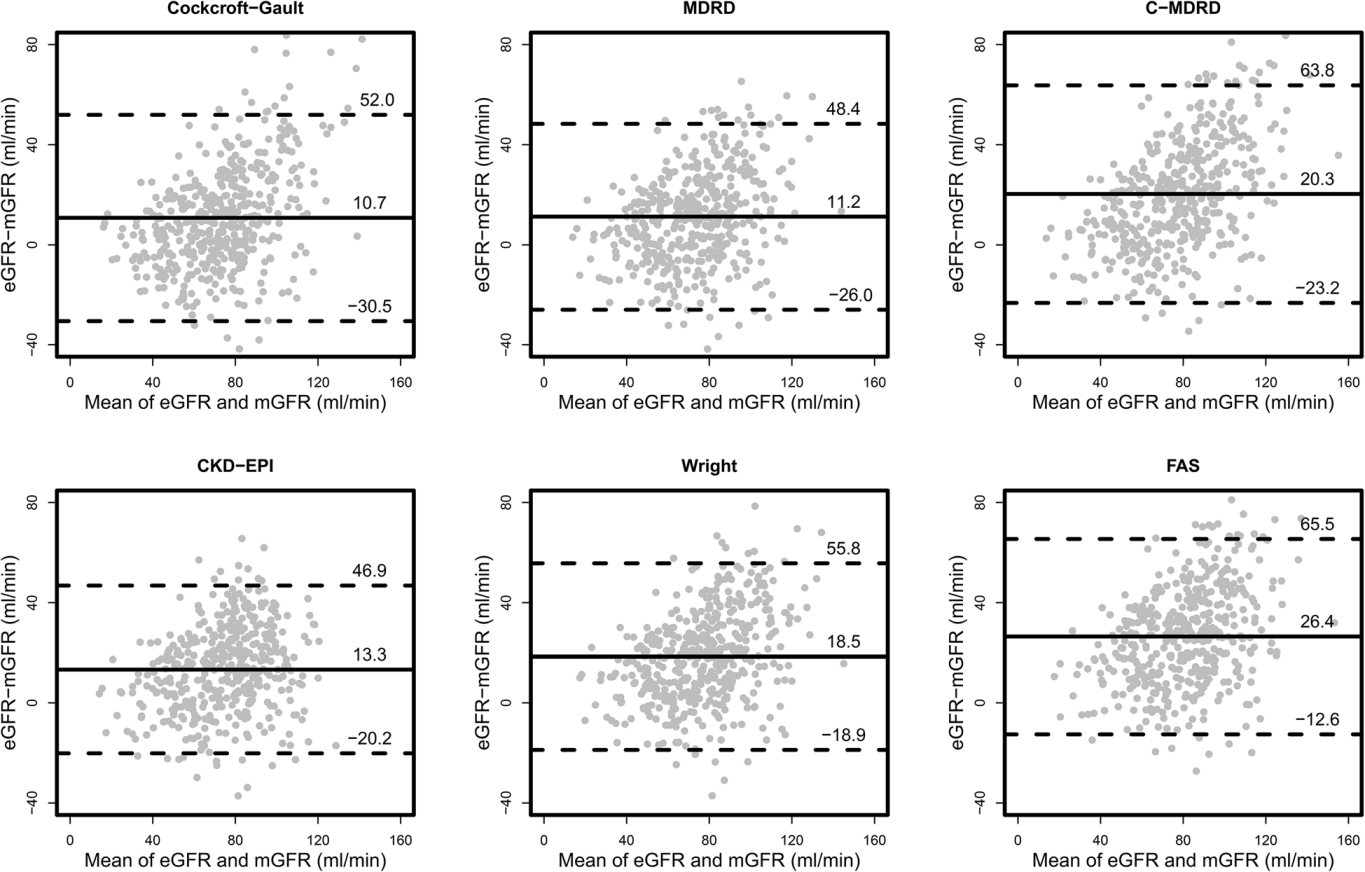
Bland-Altman plots of the estimated and measured GFR. (eGFR-mGFR) represents the difference between the estimated GFR (eGFR) and the measured GFR (mGFR). A positive difference indicates an overestimation by the equation, whereas a negative difference indicates an underestimation. The solid lines indicate the mean difference; the dashed lines indicate the lines of agreement, calculated as the mean difference ± 1.96 SD of this difference

The distribution of eGFR for the six formulae was shifted to the right, compared with the mGFR (Fig. 1). For all formulae, the eGFR tended to overestimate the mGFR. Furthermore, description of the agreement between eGFR and mGFR was shown in Bland-Altman plot (Fig. 2).

The MAPE of eGFR formulae varies from 27.8 and 34.5%, Cockcroft-Gault and MDRD formulae showed low MAPE. The absolute difference between eGFR and mGFR was greater than 30% in more than one third of the oncology patients (Table 3). CCC ranged from 0.42 to 0.61 in all formulae, reflecting low precision and accuracy. TDI ranging from 36 to 55%. MDRD formula showed lowest TDI, that is, the 90% of estimations errored from − 36 to 36% of mGFR for eGFR calculated with MDRD. Besides, the CP of MDRD formula was 35, which is highest in six formulae, indicating that more than 65% of the estimation had an error greater than ±10%.

**Table 3:** Performance of formulae

Equations	MPE	MAPE	P30	P10	CCC	TDI	CP
Cockcroft-Gault	17.8 (14.7, 20.8)^cdef^	27.8 (25.5, 30.1)^cef^	62.8 (58.3, 67.3)^cef^	24.8 (20.8, 28.8)^cf^	0.60 (0.56)	39 (41)	34 (32)
MDRD	19.2 (16.2, 22.0)^cef^	27.8 (25.5,29.9)^cef^	63.0 (58.6, 67.6)^cef^	24.3 (20.3, 28.3)^cf^	0.61 (0.57)	36 (38)	35 (33)
C-MDRD	32.3 (29.0, 35.6)^abdef^	37.5 (34.7, 40.3)^abdf^	49.7 (45.0, 54.3)^abdef^	17.0 (13.6, 20.6)^abf^	0.49 (0.46)	49 (51)	24 (23)
CKD-EPI	22.9 (20.0, 25.7)^acef^	29.2 (26.9, 31.4)^cef^	60.6 (56.0, 65.1)^cef^	21.6 (17.8, 25.5)^f^	0.60 (0.57)	36 (37)	33 (31)
Wright	30.7 (27.6, 33.6)^abcdf^	34.5 (27.6, 33.6)^abdf^	51.9 (47.4, 56.7)^abcdf^	20.2 (16.5, 24.0)^f^	0.53 (0.50)	44 (45)	26 (25)
FAS	44.0 (40.6, 47.4)^abcde^	45.9 (42.7, 49.1)^abcde^	38.0 (33.5, 42.4)^abcde^	11.7 (8.7, 14.6)^abcde^	0.42 (0.38)	55 (56)	17 (16)

Data are presented with 95% CIs. *MPE* mean percentage error, *MAPE* mean absolute percentage error; P30 and P10, percentage of patients within 30 and 10% that estimated from the measured glomerular filtration rate. *CCC* concordance correlation coefficient, *TDI* total deviation index, *CP* coverage probability, *MDRD* modification of diet in renal disease, *C-MDRD* modified MDRD Study equation for Chinese, *CKD-EPI* chronic kidney disease epidemiology collaboration, *FAS* full age spectrum. ^a^*p* < 0.05 compared with CG equation; ^b^*p* < 0.05 compared with MDRD equation; ^c^*p* < 0.05 compared with C-MDRD equation; ^d^*p* < 0.05 compared with CKD-EPI equation; ^e^*p* < 0.05 compared with Wright equation; ^f^*p* < 0.05 compared with FAS equation

Given that MDRD formula exhibited best performance among four formulae, we explored factors that would affect the accuracy of eGFR based on the MDRD formula.

### Prediction model for inaccurate eGFR calculated by the MDRD formula

**Fig. 3:**
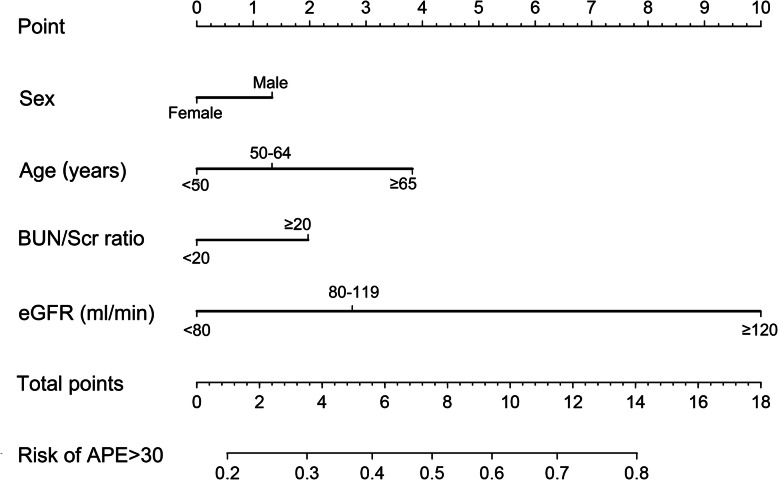
Nomogram predicting the risk of inaccurate eGFR calculated as by the MDRD formula. BMI, body mass index; BUN/Scr ratio, blood urea nitrogen to creatinine ratio; APE, absolute percentage error

Using multivariate logistic regression analysis, we noted that the poor accuracy of the MDRD was independently associated with age and eGFR level (Table 4). A nomogram was generated based on four variables, including age, sex, eGFR level and BUN/Scr ratio (Fig. 3). A higher total point scores as calculated by the sum of the assigned number of points for each predictor in the nomogram was associated with a higher likelihood of inaccurate eGFR as calculated by the MDRD formula in oncology patients. For example, a man (1.3 points) with age over 65 years (3.8 points), eGFR between 80 and 120 ml/min (2.8 points) and BUN/Scr ratio over 20 (2.0 points) would have a total of 9.9 points score, and therefore have a 63% predicted risk of inaccurate eGFR as calculated by the MDRD formula.

**Table 4:** Logistic regression analysis for factors associated with inaccurate eGFR using MDRD

	Univariate analysis		Multivariate analysis	
	OR (95%CI)	*P* value	OR (95%CI)	*P* value
Age (years)				
< 50	1.00 (reference)		1.00 (reference)	
50–64	1.17 (0.72, 1.91)	0.519	1.41 (0.81, 2.45)	0.225
≥ 65	1.68 (0.99, 2.86)	0.055	2.56 (1.31, 4.98)	0.006
Sex, male	1.36 (0.90, 2.07)	0.143	1.36 (0.85, 2.17)	0.197
BMI (kg/m^2^)				
< 20	1.00 (reference)		1.00 (reference)	
20–24.9	0.95 (0.55, 1.66)	0.868	0.76 (0.42, 1.38)	0.37
25–27.9	1.03 (0.55, 1.93)	0.916	0.78 (0.39, 1.57)	0.485
≥ 28	1.87 (0.83, 4.25)	0.133	1.34 (0.55, 3.27)	0.526
eGFR (ml/min)				
< 80	1.00 (reference)		1.00 (reference)	
80–89	1.62 (1.08, 2.43)	0.019	2.10 (1.29, 3.42)	0.003
≥ 120	6.70 (2.53, 17.74)	< 0.001	9.82 (3.47, 27.83)	< 0.001
BUN/Scr ratio				
< 20	1.00 (reference)		1.00 (reference)	
≥ 20	1.68 (1.10, 2.57)	0.016	1.53 (0.96, 2.44)	0.076
Albumin (g/L)				
< 40	1.00 (reference)		1.00 (reference)	
≥ 40	0.94 (0.63, 1.39)	0.750	1.16 (0.73,1.84)	0.519
Anemia	0.99 (0.64, 1.53)	0.962	1.09 (0.63, 1.88)	0.505
Diabetics	0.91 (0.47, 1.75)	0.769	0.83 (0.41, 1.69)	0.609
Hypertension	1.21 (0.80, 1.83)	0.364	1.06 (0.65, 1.73)	0.808
Obstructive nephropathy	0.93 (0.59, 1.46)	0.743	1.30 (0.77, 2.2)	0.323

All variables listed in the table were included in the logistic regression model. *OR* odds ratio, *95% CIs* 95% confidence intervals, *BMI* body mass index, *eGFR* estimated glomerular filtration rate, *BUN* blood urea nitrogen, *Scr* serum creatinine, *BUN/Scr ratio* blood urea nitrogen to creatinine ratio

**Fig. 4:**
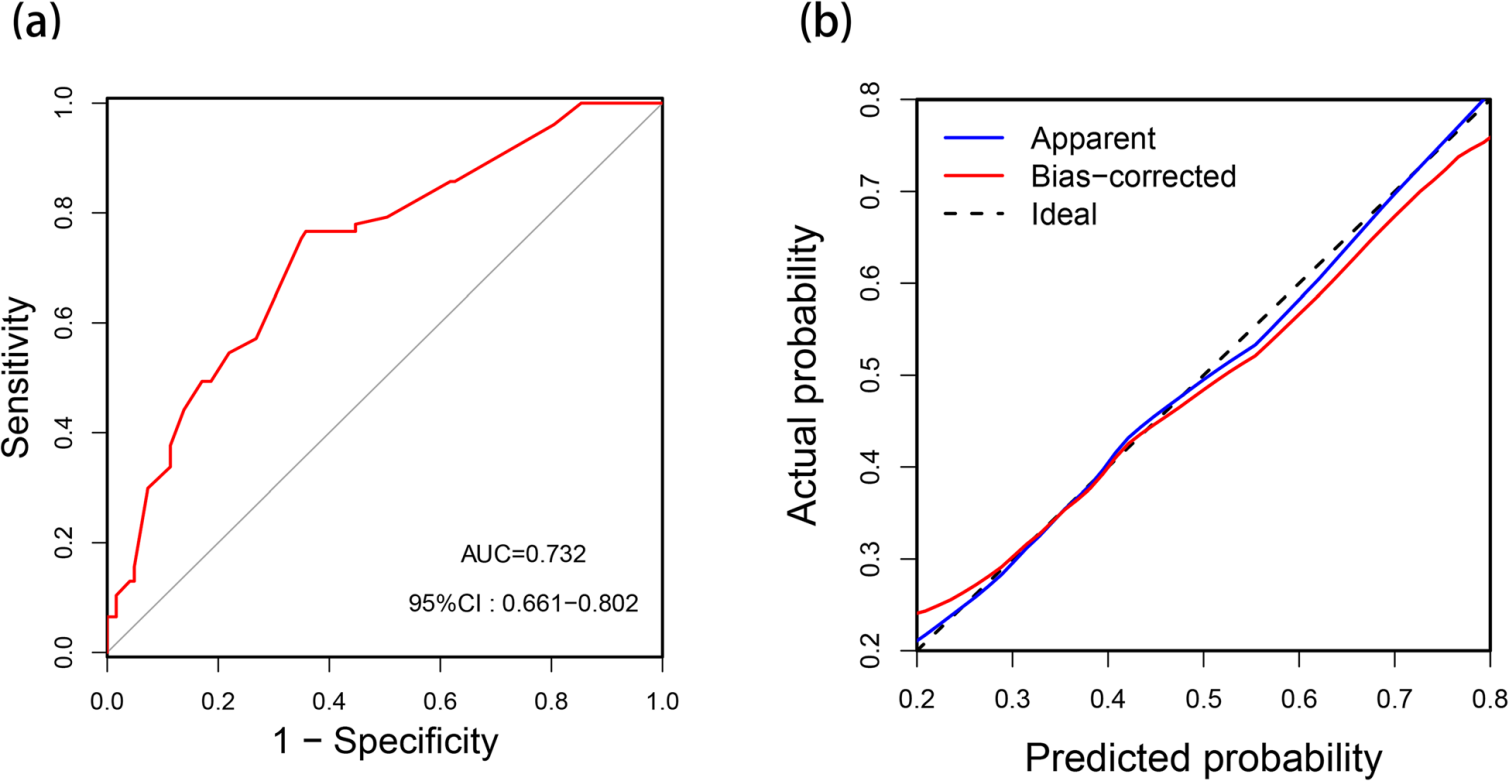
Internal validation of nomogram. **a** Receiver operating characteristic (ROC) curve. **b** Calibration curve

The predictive model had an area under curve (AUC) of 0.732 (95% CI, 0.661–0.802) after the 200 repetitions of bootstrap sample corrections. The goodness-of-fit of the nomogram was assessed by producing a calibration plot, which revealed good agreement between the predicted and observed probabilities (Fig. 4).

## Discussion

An accurate assessment of the renal function is essential in oncology patients to ensure safe prescribing of chemotherapy drugs, detecting kidney injury and assessing prognosis. Oncologists often rely on formulae to estimate the GFR on the basis of serum creatinine and other parameters. In this study, we assessed the performance of six eGFR formulae, including Cockcroft-Gault, MDRD, C-MDRD, CKD-EPI and FAS. We found that all formulae performed poorly and MDRD formula showed best agreements with mGFR. Furthermore, we found that inaccuracy of eGFR estimated using MDRD formula were more likely observed in elderly patients, as well as patients with higher eGFR. Besides, we constructed a nomogram to predict the risk of inaccuracy of eGFR.

Oncologists should pay attention to the limitation of eGFR formulae. In our study, the fraction of patients with eGFR absolute percentage error > 30% is more than one-third in all formulae. Besides, all six formulae showed positive MPE, demonstrating that these eGFR formulae tended to overestimate mGFR in Chinese cancer patients, which is inconsistence with previous studies [10, 23]. MDRD showed lowest bias and lowest agreements with mGFR, nevertheless, 90% of the estimations calculated using MDRD formula showing an error of about ±36% of mGFR. Therefore, the accuracy of eGFR formulae was unacceptable in some cases and it is inappropriate to estimate kidney function using eGFR formulae in all oncology patients. Previous studies also have criticized the performance of eGFR in estimating real renal function [24]. Oncologists should identify populations where eGFR formulae based on serum creatinine is not likely to provide an accurate estimate and thus alternative measurements of the GFR should be considered. We also assessed the modified MDRD formula for Chinese population in our study, however, C-MDRD did not perform better than original MDRD in our study. Larger population is needed to validate C-MDRD formula and further studies may focus on developing eGFR formula for Chinese cancer patients.

There are multiple possible explanations of poor performance of eGFR in Chinese cancer patients, including the lack of an ethnic factors, different serum creatinine test methods and daily changing GFR in cancer patient. Besides, oncology patients tended to have low muscle mass and reduced dietary protein intake, which would also influence the concentration of serum creatinine and thus the performance of eGFR formulae [25]. Some studies have demonstrated that the performance of eGFR formulae may be affected by age, weight and GFR [13, 14, 26].

BUN/Scr ratio may be an indicator of the accuracy of eGFR formulae. BUN/SCr ratio greater than 20 is known as a marker of pre-renal renal dysfunction [27]. Poggio et al. found that the MDRD formulae were not reliable in sick hospitalized patients, especially those with high BUN/SCr ratio [28]. However, little is known about the association of elevated BUN/SCr ratio and accuracy of eGFR formulae in oncology patients. We found that the MDRD formula was likely to be inaccurate in oncology patients with high BUN/SCr ratio. Possible explanation for our observations is that BUN/SCr ratio may rise in oncology patients with low rate of creatinine generation, and creatinine-based eGFR formulae would perform poorly accordingly.

Normal estimates of the GFR might not be actually that normal. In patients of higher eGFR, eGFR formula showed low accuracy and great degree of overestimation, which is consistent with reports from studies consisting of population with normal renal function, such as kidney donors [29, 30]. Most of the eGFR equations were derived from subjects with kidney disease, or in combined healthy/diseased populations. This may explain the overestimation of GFR in subjects with normal kidney function. Furthermore, kidney injury will be wrongly labeled as normal kidney function and the degree of renal damage will be underestimated, which encourages oncologists to make wrong decisions regarding the administration of iodinated contrast medium, employment of nephrotoxic drugs and the time to initiate renal replacement therapy. Besides, narrow therapeutic index is a pharmacokinetic characteristic of most chemotherapy agents. A well-known example of such agents is carboplatin, whose dose is adjusted by Calvert formula incorporating the GFR as an important variable [31]. As a consequence, overestimated GFR may result in overdosage of chemotherapy agents, particularly those agents which are entirely eliminated by the kidneys in unchanged active form. Ultimately, inaccurate assessment of the GFR means severe side effects, as well as increasing incidence of renal insufficiency, or even death.

An important facet of this study is that a nomogram was developed to predict the reliability of eGFR as calculated by the MDRD formula in oncology patients. The nomogram is a simple graphical prediction tool. By assigning points to the four variables, oncologists can assess the predictive risk of individuals. This provides clinically useful information and guide personalized clinical decision-making regarding whether to use accurate GFR measurements for oncology patients. Furthermore, our nomogram is constructed on the basis of readily available clinical data making it easy for clinicians to use. Internal validation indicated good performance with area under ROC of 0.732 and accurate calibration.

We acknowledge several limitations of this study. First, due to the retrospective nature of our study, there might have been selection bias and unknown confounders in the analysis. Second, we used enzymatic creatinine assay in our study, and it may lead to bias when assessed Cockcroft-Gault formula which was developed by Jaffe assay. Third, the difference of accurate GFR measurement method in our study is different from those previous studies eGFR formulae development, which may contribute to the poor correspondence between eGFR and mGFR. mGFR in our study was gained by ^99m^Tc-DTPA, which was used in development of Chinese MDRD formula. However, mGFR was determined by 24-h urine creatinine excretion in Cockcroft–Gault formula and measured by iohexol in FAS formula. Fourth, imprecision GFR measurements may lead to error between GFR estimates versus measurements. Finally, although the nomogram was validated internally by bootstrap resampling, external validation using an independent data set was required before routine use.

## Conclusions

On the basis of our findings, oncologists must be aware of the limitations of eGFR formulae when treating elderly patients, as well as patients with eGFR greater than 120 ml/min. We constructed a nomogram that could help clinicians to predict the reliability of eGFR as calculated by the MDRD formula.

## Data Availability

The datasets used during the current study are available from the corresponding author on reasonable request.

## Abbreviations

GFR: Glomerular Filtration Rate
eGFR: Estimated Glomerular Filtration Rate
Scr: Serum creatinine
MDRD: Modification of Diet in Renal Disease
C-MDRD: Modified MDRD for Chinese population
CKD-EPI: Chronic Kidney Disease Epidemiology
FAS: Full Age Spectrum
^99m^Tc-DTPA: Technetium-99 m diethyl triamine penta-acetic acid
mGFR: Measured GFR
BSA: Body Surface Area
BMI: Body Mass Index
SD: Standard Deviation
PE: Percentage Error
APE: Absolute Percentage Error
AIC: Akaike Information Criterion
ROC: Receiver Operating Characteristic
BUN: Blood Urea Nitrogen
OR: Odds Ratio
AUC: Area Under Curve
^51^Cr-EDTA: Chromium 51 Ethylene Diamine Tetra-acetic Acid
